# International Inequality and the COVID-19 Pandemic

**DOI:** 10.1007/s10272-022-1026-9

**Published:** 2022-02-12

**Authors:** Michael Dauderstädt

**Affiliations:** Bonn, Germany

**Keywords:** C82, D30, I14

## Abstract

The lockdowns and stimulus programmes that governments have adopted to fight the COVID-19 pandemic and the associated economic crisis have affected the distribution of income and production within and between countries. Considering both, current evidence indicates that the EU-wide and global inequality of disposable income did not change dramatically in 2020. However, the unequal impact on the wealth and health of people is likely to worsen income inequality in the future.

The pandemic as well as the lockdowns and stimulus programmes that governments have adopted to fight it and the associated economic crisis have affected different people, industries and countries to different degrees, with respect to the distribution of income and production. Many people lost market income as their own or their employer's business suffered from lockdown or collapsing demand. But thanks to public spending programmes, disposable income has not changed much, mainly in high income countries but also in several middle income countries like Brazil.

Some industries, notably (air) travel, accommodation, tourism and non-food retail, were affected more by lockdowns or consumers' fears than others (communication and information technology firms boomed). This sectoral bias led to different national recessions. Economies that had been strongly reliant on tourism experienced deeper crises – in Europe, Mediterranean countries suffered most. Worldwide, the economies with the steepest decline of GDP between 2019 and 2020 were Macao (-56.9%) and the Maldives (-33.2%) against a global average of -3.5%, according to World Bank data. Rich countries able to provide massive fiscal and monetary stimuli had an advantage vis-à-vis poor and already highly indebted nations.

**Michael Dauderstädt**, Freelance consultant and writer, Bonn, Germany.

This article assesses how these processes affected the distribution of income within the European Union (EU)[1] and globally.

Inequality has many dimensions and definitions. This article focuses primarily on the distribution of income. Occasionally, it considers other dimensions of welfare such as wealth or health and their unequal distribution. The concept of income used here is disposable income, i.e. market income minus taxes plus transfers received. The corresponding data is collected through household surveys, which usually deliver their numbers years later. Therefore, we often have to use GDP per capita. In order to compare values from different countries, the incomes have to be calculated in a common currency either on the base of exchange rates or purchasing power parities (PPP). Using the latter increases the income of poorer countries as their price levels are lower than in richer economies, thus showing lower levels of inequality.

Calculating the international distribution of income is tricky. In his seminal work, *Worlds Apart: Measuring International and Global Inequality*, Branko Milanovic (2005) differentiated three metrics: by country, regardless of size; by country, weighted by population but still bracketing out domestic inequality; and by person, regardless of nationality, thus considering within- and between-country inequality. The third represents the most accurate approach but requires enormous data, unavailable on a global scale for recent years. To assess the impact of the pandemic on the international distribution of income, this article provides estimates of inequality according to the second and, where possible, the third concept, which requires data on the distribution of in-

1 The section on Europe draws on Dauderstädt (2022).

come within countries and relies on the results of household surveys.

Inequality can be measured using different indicators such as the Gini coefficient, income shares or poverty rates. We mainly use the quintile ratio (or S80/S20 ratio), which compares the income shares of the richest and the poorest quintile of the population under consideration (of a country, Europe or the world).

## The heterogeneous economic impact of the pandemic across EU economies

A true picture of the EU-wide distribution of income requires the combination of both, the distribution within and between member states (Dauderstädt, 2020). Generally, within-country inequality accounts for approximately 80% of EU-wide inequality, with the rest caused by income disparities between countries. However, the evolution of EU-wide inequality is driven by the inequality between member states, as inequality within countries, on average, has not changed substantially since 2005 (see Figure 2, Eurostat curve).

The pandemic affected the economies of all member states, albeit to a different degree. For the EU27, GDP in the third quarter of 2020 was only 5% below its level in the last quarter of 2019, although it had declined by 16% during spring 2020. For the whole year, EU27 GDP was 4.7% lower than in 2019. Between 2019 and 2020, the standard variation of per capita GDP increased from €20,857 to €20,994 while average per capita GDP declined from €31,591 to €30,540 as national growth rates varied widely.

Figure 1 shows the GDP changes between 2019 and 2020 for all member states ordered by their 2019 per capita GDP. Only three countries escaped the recession: Ireland, Lithuania and Luxembourg. Among the other member states, some (mostly Nordic) countries experienced a relatively modest GDP decline of less than 2%, while in others it dropped by more than 6%. As the trend line in Figure 1 indicates, poorer countries were more likely to experience stronger recessions than richer ones. However, the eastern periphery performed well (with the exception of Croatia and Hungary), while relatively rich countries such as the UK, Italy or France experienced massive drops. The EU's southern periphery (Cyprus, Greece, Italy, Portugal and Spain) suffered most, thus aggravating already existing problems resulting from the Great Recession of 2009, the subsequent sovereign debt panic and the ill-designed austerity policies (Gräbner et al., 2020). The pandemic reinforced the previous regional pattern of catch-up growth in the EU (Dauderstädt, 2021b).

Figure 1
**Economic growth of EU member states , 2019-2020**

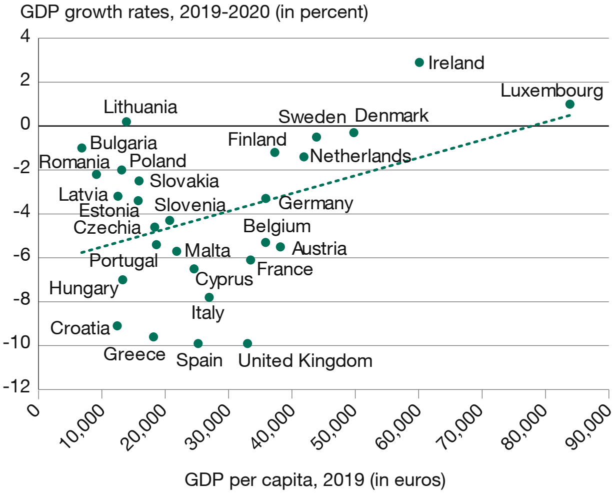

Source: Author's calculations based on Eurostat.

### Stabilised within-country income distribution

Assessing changes of the within-country inequality is difficult as the usual data sources, namely household surveys (in the case of the EU, the Statistics on Living and Income Conditions; EU-SILC), are not yet available for all countries for 2020.

In 2019, the values for the quintile ratio were 7.4 for net market income and 4.8 for disposable income on average for all 28 member states. For market income, national values varied widely, ranging from above ten (for Bulgaria, Ireland and Sweden) to five and below (for Czechia, Slovakia, Hungary and Slovenia). For disposable income, as shown in Table 1, the quintile ratio ranged between values below four (Belgium, Czechia, the Netherlands, Slovenia, Slovakia and Finland) and above seven (for Bulgaria and Romania). The difference between the value for net market income and that for disposable income indicates the effectiveness of redistribution. It is highest in Ireland and the Scandinavian countries – 7.9 in Ireland, 6.6 in Sweden and 5.4 in Denmark – while redistribution is relatively weak in Central Eastern Europe. The threshold for being at risk of poverty is defined as an income below 60% of the median equivalised disposable household income after social transfers. The at-risk-of-poverty rate is the percentage of the population at risk. The share is 21.13% on average for all countries (excluding Germany), ranging from values above 30% for Bulgaria, Greece and Romania to below 15% for Czechia and Slovakia.

Table 1

**Disposable income inequality and poverty in 2019 and 2020 in selected countries**

| | S80/S20 ratio | | | At-risk-of-poverty rate | | |
|---|---|---|---|---|---|---|
| Country | 2019 | 2020 | Change (in %) | 2019 | 2020 | Change (in %) |
| Belgium | 3.61 | 3.65 | 1.11 | 19.5 | 18.9 | -3.08 |
| Bulgaria | 8.10 | 8.01 | -1.11 | 32.8 | 32.1 | -2.13 |
| Czechia | 3.34 | 3.34 | 0 | 12.5 | 11.9 | -4.80 |
| Denmark | 4.09 | 4.00 | -2.20 | 16.3 | 15.9 | -2.45 |
| Germany | 4.89 | 6.47 | 32.31 | 17.4 | 24.0 | 37.93 |
| Estonia | 5.08 | 5.03 | -0.98 | 24.3 | 23.3 | -4.12 |
| Greece | 5.11 | 5.15 | 0.78 | 30.0 | 28.9 | -3.67 |
| Spain | 5.94 | 5.77 | -2.86 | 25.3 | 26.4 | 4.35 |
| France | 4.27 | 4.48 | 4.92 | 17.9 | 18.2 | 1.68 |
| Croatia | 4.76 | 4.61 | -3.15 | 23.3 | 23.2 | -0.43 |
| Cyprus | 4.58 | 4.31 | -5.90 | 22.3 | 21.3 | -4.48 |
| Latvia | 6.54 | 6.27 | -4.13 | 27.3 | 26.0 | -4.76 |
| Lithuania | 6.44 | 6.14 | -4.66 | 26.3 | 24.8 | -5.70 |
| Luxembourg | 5.34 | 4.99 | -6.55 | 20.6 | 20.9 | 1.46 |
| Hungary | 4.23 | 4.30 | 1.65 | 18.9 | 17.8 | -5.82 |
| Malta | 4.18 | 4.69 | 12.20 | 20.1 | 19.0 | -5.47 |
| Netherlands | 3.94 | 4.04 | 2.54 | 16.5 | 16.3 | -1.21 |
| Austria | 4.17 | 4.11 | -1.44 | 16.9 | 17.5 | 3.55 |
| Poland | 4.37 | 4.07 | -6.86 | 18.2 | 17.3 | -4.95 |
| Portugal | 5.16 | 4.99 | -3.29 | 21.6 | 19.8 | -8.33 |
| Romania | 7.08 | 6.62 | -6.50 | 31.2 | 30.4 | -2.56 |
| Slovenia | 3.39 | 3.32 | -2.06 | 14.4 | 15.0 | 4.17 |
| Slovakia | 3.34 | 3.03 | -9.28 | 16.4 | 14.8 | -9.76 |
| Finland | 3.69 | 3.72 | 0.81 | 15.6 | 16.0 | 2.56 |
| Sweden | 4.33 | 4.12 | -4.85 | 18.8 | 17.9 | -4.79 |
| Average | 4.80 | 4.77 | -0.38 | 20.98 | 20.70 | -0.91 |
| Average without Germany | 4.79 | 4.70 | -1.74 | 21.13 | 20.57 | -2.53 |

Note: The highlighted cells indicate an increase in disposable income inequality and at-risk-of-poverty rates, respectively, in 2020.

Source: Eurostat TESSI180 and ILC_PEPS01, November 2021.

Looking at the latest available evidence on income, the Eurostat data for the 25 countries that have already provided figures for 2020 (see Table 1) hardly shows any major changes. Disposable income inequality has only slightly changed in almost all countries, with only eight countries showing an increase (highlighted cells). At-risk-of-poverty rates have actually declined in all but seven countries (highlighted cells), albeit by less than one percent. The dramatic outlier is Germany, where the quintile ratio increased by 32% and the at-risk-of-poverty rate by 37%. These rises are caused by a change in the data collection method of Destatis, the German statistical office, which make the data incomparable with previous years. On average, for the whole EU (unweighted, excluding Italy and Ireland), the quintile ratio declined by 0.38% or, more plausibly, 1.74% (excluding Germany) and the at-risk-of-poverty rate declined by 0.91% or 2.53% (excluding Germany).

For Germany, where data from different years is no longer comparable, and Italy, where EU-SILC data are not yet available, country-specific studies confirm this assessment.

*Germany*

According to Hövermann and Kohlrausch (2020), people with lower incomes reported higher losses of earnings than those with higher incomes. Analysing the German low-wage sector, Schulten (2020) expects poverty to rise. The Hans Böckler Foundation (2020) cited polling data that points to an increase in inequality in Germany. Pieper et al. (2021) expect a rise in poverty, affecting in particular poor pensioners, who do not benefit from public COVID-19 aid programmes. All these authors question whether the short-time working compensation paid by the state (and employers) will sufficiently stabilise incomes. A study by researchers from the German Institute for Employment Research finds that low-wage earners are hit harder by unemployment than other wage groups (Buch et al., 2021).

Other studies expect no changes in the distribution of income. The results of a simulation study conducted by Bruckmaier et al. (2020; see Figure 1) point to gross incomes declining by 3% on average and, for the different income deciles, between 4.3% for the first decile and 2.8% for the tenth decile, implying a slight increase in inequality in market incomes. However, these findings are quite different for disposable income: Here, the average decline is just 0.1%, with incomes rising in the lower deciles and declining in the higher ones (Bruckmaier et al., 2020; see Figure 2). Research by the German Economic Institute suggests similar results, with strong declines in market incomes in the lower income deciles being largely compensated, thus stabilising disposable incomes (Beznoska et al., 2021). Grabka (2021) posits that the pandemic slightly reduced income inequality in Germany.

The picture for Germany is ambiguous: While market income inequality is likely to have increased in 2020, the distribution of disposable incomes might not have changed much. Given the size of Germany's income support spending, a dramatic rise in inequality is highly unlikely, also in the light of the development in most other European countries. Unfortunately, the change in the data collection method by the Federal Statistical Office of Germany prevents a reliable assessment.

*Italy*

Looking at Italy, a study by researchers at the Bank of Italy on the impact of the pandemic on labour incomes shows that the market incomes of lower-paid workers decreased more than those of high earners. Due to the state support schemes, however, the resulting distribution of net disposable income hardly changed (Carta and De Philippis, 2021). A larger survey (Cánto Sánchez et al., 2021) of four countries (Belgium, Italy, Spain and the UK) found that inequality (Gini) increased only in Italy. Lower income strata were less affected than higher ones due to welfare programmes. Clark et al. (2020) use panel data of another survey and posit that the inequality of disposable income fell during the first nine months of 2020 in France, Germany, Italy, Spain and Sweden. In sum: In Europe, the within-country distribution of disposable income has hardly changed in the wake of the pandemic due to government programmes.

## COVID-19 and EU-wide inequality

As can be seen in the lowest curve (referred to as "Eurostat") in Figure 2, average inequality, measured by the quintile ratio, has always oscillated around 5 (no data yet available for 2020). This curve is the EU-wide value given by Eurostat as the weighted (by population) average of the national values. It is misleading, however, as it neglects the income disparities between countries (Atkinson, 2010; Dauderstädt, 2020). For example, EU-wide inequality jumped in 2007 with the accession of Bulgaria and Romania. It then declined (as between 2005 and 2007) due to strong growth in Eastern Europe. The financial crisis stopped and partially reversed that decline. Following a decade of stagnation, cohesive growth resumed in 2017 but slowed again in the wake of the pandemic.

As shown above, both within-country and between-country inequality have not changed much, and the impact of the pandemic on EU-wide inequality, as measured by the S80/S20 ratio, has been weak so far (Dauderstädt, 2021a). The main reason for that is that the effect of the large differences between national growth rates (see Figure 1) has been weak, as the hardest hit southern periphery represents the lower middle income group of the EU, with per capita incomes around €20,000. The low income group on the eastern periphery (with per capita incomes below €20,000) and the high income group of the north-western centre (with per capita incomes above €30,000) both suffered less on average, albeit with very diverse national performances.

Figure 2

**EU-wide inequality: Income quintile share ratio (S80/S20), 2005-2020**

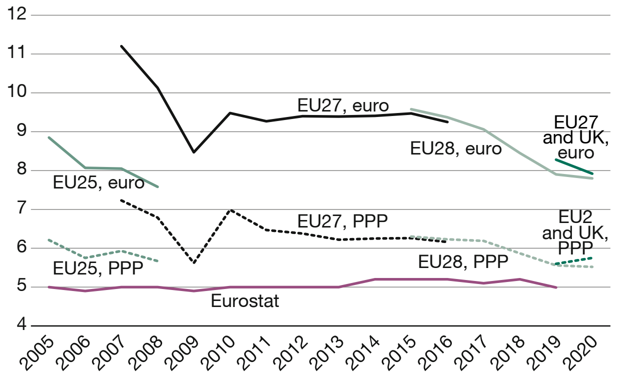

Note: Data for Ireland and Italy are still lacking for 2020; average quintile incomes for Ireland and Italy have been estimated using the 2019 data adjusted by the growth rates of income and population of the whole economy (thus assuming an equal distribution of growth between all quintiles).

Source: Dauderstädt (2021a) and Eurostat; November 2021.

Figure 2 shows two values for the years 2019 and 2020. The lower value EU28 (calculated in spring 2021, when EU-SILC data were not yet available) assumes no changes in the within-country distribution of income and its slight decline results from the evolution of between-country inequality (see Dauderstädt, 2021a). The slightly higher values for 2019 and 2020 represent the EU27 after Brexit and are based on the EU-SILC data (as in Table 1). They include the high values for Germany which are no longer comparable with previous years. If one replaces these values for Germany by estimates that assume an equal distribution of income growth among quintiles, the resulting estimates are close to but even slightly lower than our spring 2020 estimates. At the end of the day, the pandemic seems to have only slowed the decline in inequality (or reversed it somewhat, albeit only at exchange rates, if one accepts the new German data at face value).

The picture is different for wealth inequality: due to the loose monetary policy of the ECB and other central banks, the value of many assets, in particular stocks and property, has increased, benefiting richer households. Savings increased dramatically during the COVID-19 pandemic crisis. In Germany, the savings rate increased in 2020 to 16.3% of disposable income – an increase of

5.4 percentage points compared to 2019. Financial assets reached €6.7 trillion at the end of the third quarter of 2020, up by €108 billion (or 1.6%) compared to the second quarter (Bundesbank, 2021). But higher wealth inequality does not translate immediately into higher income inequality, as returns on capital in a zero interest rate economy are small.

## The pandemic's impact on the global distribution of income

Contrary to the EU case, data regarding the within-country distribution of income in 2020 are not available on a global scale. Even for the years before 2019, the World Bank (World Development Indicators) provides quintile income shares for less than half of the global population. Thus, an estimate of the current true global inequality according to Milanovic's third concept is extremely difficult. We focus on the second concept, which ignores the within-country distribution. This approach turned out to be sensible with regard to the EU, where national inequalities hardly changed due to government policies, but might be less accurate on a global scale. For the US, a study by JPMorgan Chase & Co. (2021) showed that low incomes increased more during the pandemic than high incomes. In developing countries, the poor are often working in the informal economy where they are not benefiting from compensating policies such as job retention schemes. Increasing poverty under these circumstances might become statistically visible but only a year or two from now.

In February 2021, Deaton (2021) published a provocative analysis positing, contrary to widespread belief, that global inequality had declined during the pandemic. The GDP of high-income countries declined more than that of poorer countries, mainly because death rates in 2020 were higher in richer countries. But this effect disappears when the countries are weighted by population. In 2021, many poorer countries, in particular India, experienced new waves of COVID-19 infection, with a high number of deaths. The International Monetary Fund (2021) forecasts for 2021 show higher economic growth for emerging and developing economies than for advanced ones (6.8% versus 5.1%), probably due to the stellar performance of China. Their share of global GDP (at PPP) increased from 56.5% in 2018 to 57.8% in 2021, while the advanced countries' share declined from 43.5% to 42.2%. But soon, emerging economies might face more obstacles to catch-up growth, such as higher interest rates, declining demand for commodities from China, weaker growth of world trade and new waves of the coronavirus.

To approximate an adequate estimate of inequality defined by Milanovic's second concept, the global quintile

Figure 3

**Global income inequality: Income quintile share ratio (S80/S20), 2016-2020**

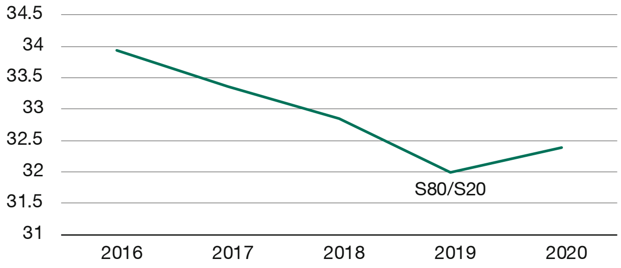

Source: Author's calculations based on World Bank.

(S80/S20) ratio compares the income of the richest fifth of the world's population with that of the poorest fifth. To calculate the global value, GDP and population figures from the World Development Indicators database of the World Bank allow countries covering 98% of the global population to be ranked by per capita income. Including as many of the poorest and richest countries as necessary to get one fifth of the global population (approximately 1.5 billion) in each case, in 2020 the income of the poorest quintile amounted to about US $1.7 trillion (of a total world GDP of about US $80.8 trillion), while the richest fifth took in US $55 trillion. This results in an extremely high S80/S20 ratio of 32.4.

Figure 3 shows the respective values for the 2016-2020 period. Global inequality (measured this way) declined until 2019 but the pandemic reversed that trend, albeit in a minor way (not even returning to the level of 2018).

It remains to be seen how the more severe impact of the pandemic in 2021 on poorer, less vaccinated countries will change that picture. Neither Deaton's optimism nor the exaggerated fears of many well-intentioned observers (e.g. Inequality.org, 2021) seems, however, to be justified by the available data. Even the most famous critics of inequality, Thomas Piketty and his colleagues, estimate that global inequality declined between 1980 and 2020 (Chancel and Piketty, 2021). Ferreira (2021) shows that estimates regarding the development of global inequality depend often on the chosen metric.

The indicator calculated here represents global inequality according to Milanovic's second concept. "True" inequality (third concept), is certainly higher because the income of the rich in poor countries lifts their overall per capita income (second concept). An estimate by Dauderstädt and Keltek (2011) gave a value of 50 for the global quintile ratio in 2005, considering within-country as well as between-country inequality. On the Gini coefficient, which ranges from 0 for total equality to 1 for total inequality, Milanovic (2013) estimated the values for his first and second concepts at about 0.5 and for his third concept at 0.7 for 2013. Both estimates indicate that true inequality – whose assessment is difficult and, due to poor data availability, only possible years later – is about 50% higher than concept-two inequality.

When we calculate poverty in the same way – considering whole countries while neglecting their internal distribution of income – the results confirm the trend shown in Figure 3. Using World Bank poverty definitions (US $1.90 or US $3.20 a day), we see a decline in incidence until 2019. The poverty reduction has been slow for the poorest (living on less than US $1.90 a day) but significant for the group below (US $3.20), falling from above 14% of the world's population in 2016 to 8.26% in 2019. The same is true if we take 60% of the global median income (about US $4,300 in 2020) as the poverty threshold (that is US $2,580 or about US $7 a day). The proportion falling below had declined from 43.7% in 2016 to 40.7% in 2019. On all three measures, poverty increased in 2020, though by less than one percentage point. But this still implies that the pandemic crisis added tens of millions of people to the global poor.

Wealth will have become more concentrated due to the asset price inflation triggered by the extremely loose monetary policies adopted by the major central banks. In spite of the dramatic recession in the second quarter of 2020, stock markets recovered quickly and achieved new highs in 2021, while house prices increased dramatically. As Oxfam research (Berkhout, 2021) reports, the wealth of the world's richest ten billionaires increased by $540 billion between March and December 2020. In the medium to long run, these developments are likely to increase income inequality, through the incomes that rich asset owners derive as rents.

## Outlook: Alarming inequalities

Changes in income and wealth might not be the most harmful (if at all) effects of the pandemic. COVID-19 affected the current and future well-being, in particular health, of many people to very different degrees. These other inequalities are exacerbated by the inequalities of income and wealth and might, in the medium and long term, reinforce the economic inequalities of income and wealth.

Even in rich Europe, the pandemic is exacerbating the well-known inequality in life expectancy which is, on average, years higher for the rich than for the poor. The poor tend to have a higher incidence of pre-existing conditions. They are also more likely to be infected because of crowded housing and the fact that low-paid workers are less likely to work from home (OECD, 2020). As regards housing, more than 25% of households in Hungary, Latvia, Poland and Slovakia live in overcrowded conditions that make infection more likely (OECD, 2020). There are not yet many studies assessing this connection in European countries. Research has identified a correlation between, on the one hand, infection and mortality, and, on the other hand, lower income and deprivation in the US and to a lesser degree in the UK (Wachtler et al., 2020). Given the emerging risk of long COVID-19, the poor might suffer more often from lasting effects reducing their productivity, employability and income, with corresponding repercussions in social protection systems. Furthermore, people suffering from financial insecurity and unemployment are more likely to be afflicted by depression and other mental health problems (OECD, 2021).

Children from poorer families have less access to digital forms of learning and communication and are usually more reliant on personal care and teaching by professionals, which their parents are often unable to provide. Children from migrant families have greater difficulties learning the languages of their host countries when deprived of opportunities to meet and communicate with native speakers (Ravens-Sieberer et al., 2021). As educational outcomes already depend overwhelmingly on the educational background of the parents, such disparities are likely to increase. In the medium and long run, educational disparities translate into professional, career and income disparities that will become visible decades later (Engzell et al., 2021).

During the lockdowns, women have had to increase unpaid care work much more than men (European Institute for Gender Equality, 2021), thereby reducing paid work and probably entailing lost career opportunities. Still often reflecting traditional role models, the division of labour within families between women and men might disadvantage women further, impeding their participation in the formal labour market (Rubery and Tavora, 2020).

Worldwide, the poor have been more likely to be infected and to die from COVID-19, due to existing health issues, worse living conditions and work that could not be done from home, even in high income countries. The number of life years lost per 100,000 people in 2020 appears to be higher in richer than in poor countries (Ferreira, 2021). But poor countries have weaker healthcare systems and higher mortality, which often will not be traced to the pandemic by the authorities although high excess mortality rates clearly indicate it. And the global population has been vaccinated to an extremely unequal degree, with very low rates of vaccination in most developing countries. Given these weaknesses and the limited systems of social protection in the poorer nations of the world, it is likely that the pandemic will increase within-country inequality and poverty in low- and middle-income countries.
